# Association between triglyceride high-density cholesterol-glucose body index and 28-day mortality in critically ill patients: a multicenter observational study in China

**DOI:** 10.1016/j.aicoj.2026.100173

**Published:** 2026-09-30

**Authors:** Xiaojun Pan, Yueming Xiao, Hangxiang Du, Zhenliang Wen, Hui Li, Chengchen Yang, Wenzhe Li, Sisi Huang, Yizhu Chen, Lidi Zhang, Tao Wang, Yongan Liu, Sheng Zhang, Dechang Chen, Jiao Liu

**Affiliations:** Department of Critical Care Medicine, Ruijin Hospital, Shanghai Jiao Tong University School of Medicine, No.197 Ruijin 2nd Road, Shanghai 200025, China

**Keywords:** TyHGB, Critical illness, Observational cohort study

## Abstract

**Background:**

The triglyceride high-density lipoprotein cholesterol-glucose body mass index (TyHGB) is a novel composite indicator integrating triglyceride, high-density lipoprotein cholesterol, fasting glucose and body mass index and may provide a more comprehensive assessment of systemic metabolic status than current indices. This multicenter landmark cohort study aimed to determine the association between TyHGB measured at the landmark time and 28-day mortality in patients present in the intensive care units (ICUs) at that time and to evaluate its potential utility as a prognostic marker.

**Methods:**

This multicenter observational cohort study included 1649 patients from 113 ICUs in China. The association between TyHGB and 28-day mortality was evaluated using three sequentially adjusted logistic regression models with restricted cubic splines: Model 1 adjusted for demographics; Model 2 further adjusted for disease severity and organ dysfunction; Model 3 additionally adjusted for therapeutic interventions. Subgroup analyses with interaction tests were conducted. Model performance was assessed by discrimination (AUC), calibration (Brier score and calibration slope) and internal validation via bootstrap resamples.

**Results:**

Restricted cubic spline analysis demonstrated a positive association between TyHGB and 28-day mortality across fully adjusted models (P < 0.05). Subgroup analyses identified a statistically significant interaction with diabetes (P for interaction = 0.011). In the overall cohort, adding TyHGB to the base model improved AUC from 0.731 to 0.745 (ΔAUC = +0.014, P = 0.043), with significant improvements in reclassification (NRI = 0.328, P < 0.001; IDI = 0.012, P = 0.009) and model fit (LRT P < 0.001). Among diabetic patients (N = 401), TyHGB significantly improved AUC from 0.673 to 0.723 (ΔAUC = +0.050, P = 0.049; NRI = 0.515, P < 0.001; IDI = 0.041, P = 0.022). In contrast, no significant improvement was observed in non-diabetic patients (ΔAUC = +0.009, P = 0.152).

**Conclusion:**

Among patients present in participating ICUs at the landmark time, higher TyHGB was associated with subsequent 28-day mortality. However, adding TyHGB to the baseline risk prediction model led to only small improvements in overall predictive performance. Findings in diabetic patients are exploratory and require further validation.

## Introduction

The global burden of critical illness has been increasingly recognized. According to a 2023 World Health Assembly resolution commentary, an estimated 30–45 million adults suffer from critical illness annually worldwide [1]. Metabolic dysregulation is closely linked to disease progression and clinical outcomes in critical illness [2]. As one of the most recognized metabolic abnormalities, hyperglycemia occurs in approximately 40–60 % of critically ill patients and is independently associated with increased mortality [3]. Beyond glucose abnormalities, alterations in lipid metabolism are also frequently observed. Among septic patients, reduced circulating lipoprotein levels have been reported in approximately 40% of patients and are associated with greater disease severity and poorer outcomes [4]. Furthermore, systemic cholesterol concentrations are inversely associated with mortality in critically ill populations [5,6]. Altered lipid metabolism therefore represents another important component of critical illness-associated metabolic dysfunction.

In response to critical illness, extensive metabolic remodeling occurs in muscle and adipose tissue and thus profoundly influence the systemic metabolic landscape, characterized by insulin resistance, hyperglycemia and hypertriglyceridemia [7]. Importantly, these abnormalities frequently coexist rather than occur independently, suggesting that integrated metabolic assessment may provide additional prognostic information beyond individual biochemical parameters. From a clinical perspective, glucose control has long been a focus of critical care research. However, blood glucose level alone appears insufficient to fully capture the complexity of metabolic derangements in this population as no benefit was observed in intensive glucose control group in multiple recent studies [[8], [9], [10]]. These findings have prompted the development of composite indices that comprehensively reflect glucose metabolism under stress conditions. Indices reflecting glucose dynamics, such as glycemic variability and the stress hyperglycemia ratio, have demonstrated prognostic value beyond static glucose measurements [11,12].

Besides, insulin resistance represents another key dimension of metabolic dysfunction. The physiological stress response to critical illness, characterized by elevated catecholamines, cortisol, and pro-inflammatory cytokines, profoundly disrupts insulin signaling and promotes a state of acquired insulin resistance [13]. The triglyceride-glucose (TyG) index, a simple surrogate marker of insulin resistance derived from fasting triglyceride and glucose levels, has shown associations with mortality across various ICU subpopulations [11,14,15]. However, the TyG index does not account for adiposity, which is closely linked to insulin resistance and metabolic syndrome.

The triglyceride high-density cholesterol-glucose body index (TyHGB) was developed as a refined extension of TyG, integrating high-density lipoprotein (HDL) and body mass index (BMI) to capture the additional contribution of adiposity and lipid metabolism to metabolic dysfunction [16]. Reduced HDL levels have been associated with mortality in critically ill patients [5]. Moreover, the long-lasting debate on the obesity paradox in critical illness suggested the potential role of BMI [7]. Emerging evidence has supported its predictive value in diabetes mellitus and non-alcoholic fatty liver disease (NAFLD) [[17], [18], [19]], while its potential role in critically ill patients has not been explored.

Clinically, the potential value of TyHGB extends beyond identifying a statistical association with mortality. Current ICU prognostic assessment largely relies on physiological derangements and established organ dysfunction, whereas metabolic abnormalities also influence disease trajectories. By integrating glucose regulation, lipid metabolism and adiposity-related information, TyHGB may provide complementary insights into the metabolic adaptability and resilience of critically ill patients. Moreover, as it is derived from routinely collected clinical parameters, TyHGB has the potential advantages of simplicity and accessibility across diverse ICU settings. Therefore, TyHGB may be a candidate marker for assessing metabolic status and complementing existing severity-based prognostic models.

Nevertheless, whether TyHGB is associated with clinical outcomes in critical care settings has not been explored. Importantly, in routine ICU practice, clinicians must assess prognosis of patients whose duration of ICU stay varies widely. Whether TyHGB retains prognostic value when measured at a single time point during ICU stay and whether it can inform short-term risk assessment for the current ICU population has not been established. Therefore, we conducted a multicenter landmark cohort study involving 113 ICUs in China. The aim was to determine whether TyHGB, measured at a single landmark time, is associated with 28-day mortality in patients present in the ICU at that time and to evaluate its potential utility as a point-in-time prognostic marker for the current ICU population.

## Methods

### Study design and participants

This was a multicenter observational study conducted across 113 ICUs in mainland China to evaluate the association between TyHGB and 28‑day mortality in critically ill patients present at the landmark time (Figure S1). The study was designed as a landmark point-prevalence cohort. All adult patients present in the participating ICUs at the landmark index time (8:00 AM on September 15th, 2021) were enrolled. Baseline clinical characteristics and laboratory parameters were collected within the standardized sampling window (6:00 AM to 12:00 PM) on the index date to calculate the TyHGB index. No exclusion criteria were applied except for age <18 years, documented limitations of medical treatment and data missing for age, gender, height or weight. All eligible patients were followed up for 28 days starting from the landmark index time (September 15th, 2021, 8:00 AM) to assess the primary outcome of 28-day all-cause mortality post-index time. The study was approved by the Research Ethics Committee of Ruijin Hospital, Shanghai Jiao Tong University School of Medicine (NO. 2020-201). All participating centers obtained local ethics approval or filed the central ethics approval with their respective institutional review boards in accordance with local regulations. Written informed consent was obtained from all participants or their legally authorized representatives.

### Definitions

#### Triglyceride-glucose index (TyG)

The TyG index was calculated using the formula: TyG index = ln[ (TG × 88.6) × (glucose × 18) / 2 ], where TG and glucose are in mmol/L [20].

#### Triglyceride high-density cholesterol-glucose body index (TyHGB)

The TyHGB index was calculated using the formula: TyHGB = TG / HDL + 0.7 × glucose (mmol/L) + 0.1 × BMI (kg/m²) [20]. The component variables were measured using routine clinical laboratory assays according to local laboratory protocols at each participating center. Before calculating TyHGB, extreme observations in TG, HDL, glucose and BMI were winsorized at the 0.5th and 99.5th percentiles to minimize the influence of extreme values while preserving the original data structure.

### Data collection

Clinical and laboratory data were systematically extracted from the electronic medical record system using a standardized electronic case report form (CRF). The dataset comprised three functional modules: (1) Baseline Characteristics: Demographic data (age, gender, BMI), admission source and pre-existing comorbidities. Disease severity was quantified using the Acute Physiology and Chronic Health Evaluation II (APACHE II) and Sequential Organ Failure Assessment (SOFA) scores calculated. (2) Metabolic and Laboratory Profiles: Comprehensive laboratory examinations were recorded from 6:00 AM to 12:00 PM, including blood cell counts, inflammatory markers (C-reactive protein, procalcitonin) and organ function indices (creatinine, BUN, ALT, GGT, albumin and lactate). Crucially, lipid and glucose profiles were collected to calculate the TyG index and TyHGB according to established formulas. (3) Clinical Outcomes: The primary endpoint was 28-day all-cause mortality. Survival status was verified through hospital records. For patients discharged within 28 days, outcomes were ascertained via telephone follow-up.

### Statistical analyses

Descriptive statistics were performed on the original observed data. Continuous variables were expressed as means ± standard deviation (SD) for normally distributed data, and as median with interquartile range (IQR) for non-normally distributed data. Normality was assessed using the Shapiro-Wilk test. Categorical variables were presented as frequencies and percentages. To evaluate differences between outcome groups (survivors vs. non-survivors), the independent-sample t-test or the Mann-Whitney U test (Wilcoxon rank-sum test) were applied according to the distribution. Categorical data were compared using the Chi-square test or Fisher’s exact test as appropriate.

The associations between TyHGB and 28-day mortality were examined using multivariable logistic regression with restricted cubic splines (RCS) to accommodate potential nonlinear dose-response relationships. Three sequentially adjusted models were constructed: Model 1 accounted for demographic factors (age, gender) and diabetes. Model 2 additionally adjusted for disease severity and organ dysfunction, including APACHE II score, lactate, creatinine, albumin and total bilirubin, variables known to reflect mortality risk in critically ill patients. Model 3 further adjusted for ICU interventions (insulin, nutrition, corticosteroids) and pre-index ICU length of stay to assess the robustness of our findings to treatment-related metabolic influences. Nonlinearity was tested using the Wald statistic for the spline terms, with a two‑degree‑of‑freedom chi‑square test. Subgroup analysis were then performed in Model 2. If the non-linearity did not exist, logistic regression analysis would be performed. To account for multiple testing and avoid false-positive subgroup differences, we strictly prioritized the evaluation of interaction P values over within-subgroup statistical significance. Subgroup analyses were considered exploratory and subgroup differences were only inferred when the interaction test reached statistical significance.

To evaluate the incremental predictive value of TyHGB, we compared models with and without each metabolic index using AUC, net reclassification improvement (NRI) and integrated discrimination improvement (IDI). The baseline model (Model A) included age, gender, APACHE II, lactate, and diabetes. Differences in AUC were compared using DeLong's method with the standard error of paired AUC differences computed using the covariance-based formula. The continuous NRI and IDI were calculated following the definitions by Pencina et al. [21]. The optimal cutoff was determined by the Youden index, with sensitivity, specificity, PPV and NPV reported accordingly. Calibration was assessed using the Brier score and calibration slope. Internal validation was performed with 500 bootstrap resamples to obtain optimism-corrected estimates. Subgroup analyses by diabetes status are presented as exploratory findings requiring validation.

Missing data were handled using multiple imputation by chained equations (MICE) with 50 imputed datasets (missing rate in Supplementary Table S1). The imputation model included all variables used in the regression models, including the outcome variable (28-day mortality), components of exposure variable (glucose, BMI, TG and HDL) and other covariates included in Models 1−3. Pre-index ICU length of stay was categorized as <3, 3–<7 and ≥7 days. Continuous variables were imputed using predictive mean matching, while categorical variables were imputed using appropriate regression models. Rubin’s rules were applied to combine estimates and standard errors across imputed datasets. To assess the robustness of our findings to missing data assumptions, a complete-case sensitivity analysis was performed on patients with complete data for all variables included in the imputation model. Baseline characteristics of the complete-case cohort and the pooled imputed cohort were compared using standardized mean differences (SMD), with values <0.1 indicating negligible imbalance. (Supplementary Table S2).

All statistical analyses were performed using R (version 4.5). Missing data were handled by multiple imputation via the mice package. Restricted cubic splines, model calibration and internal validation were implemented using the rms package. AUCs were calculated using the pROC. Two-tailed p < 0.05 was considered to indicate statistical significance.

## Results

### Characteristics of enrolled patients

The baseline characteristics and variables for the study day are presented in Table 1. A total of 1649 patients were enrolled for analysis, including 1424 survivors and 225 non-survivors. Among the 105 patients excluded due to missing data, 5 had missing age, 7 had missing gender, 98 had missing height and 87 had missing weight. Because some patients had missing values for more than one variable, the sum of these counts exceeds 105. No patients were excluded for age <18 years or for documented limitations of medical treatment. Non-survivors were older than survivors (median 67 vs 62 years; P < 0.001) and have higher APACHE II, SOFA, nutrition risk screening (NRS) 2002 and Charlson score (all P < 0.001).

**Table 1:** Baseline characteristics of the patients.

	Survivor (n = 1424)	Non-survivor (n = 225)	*p* value
Age (yr)	62.00 [50.00, 72.00]	67.00 [57.00, 75.00]	<0.001
Height (cm)	168.00 [160.00, 173.00]	168.00 [160.00, 173.00]	0.954
Weight (kg)	65.00 [55.00, 70.00]	65.00 [55.00, 70.00]	0.648
BMI	22.86 [20.68, 24.97]	22.89 [20.48, 25.10]	0.602
APACHE Ⅱ	14.00 [9.00, 19.00]	19.00 [15.00, 26.00]	<0.001
SOFA	5.00 [3.00, 7.00]	9.00 [6.00, 13.00]	<0.001
NRS 2002	3.00 [3.00, 5.00]	4.00 [3.00, 6.00]	<0.001
Charlson score	2.00 [1.00, 3.00]	3.00 [1.00, 4.00]	<0.001
Gender			0.502
Male	935 (65.7)	142 (63.1)	
Female	489 (34.3)	83 (36.9)	
Source of patients			0.339
Emergency	561 (39.4)	85 (37.9)	
Surgery	501 (35.2)	72 (32.1)	
Medical	361 (25.4)	67 (29.9)	
Comorbidities			
Diabetes	334 (23.5)	67 (29.8)	0.049
Cardiovascular	466 (32.7)	96 (42.7)	0.004
Cerebrovascular	331 (23.2)	56 (24.9)	0.648
Respiratory	156 (11.0)	32 (14.2)	0.187
Solid Tumor	155 (10.9)	25 (11.1)	1
Hematological	31 (2.2)	16 (7.1)	<0.001
Laboratory tests			
White blood cells (10^9/L)	9.55 [6.97, 13.16]	11.95 [8.18, 16.03]	<0.001
Hemoglobin (g/L)	96.00 [80.00, 111.50]	85.00 [73.00, 100.00]	<0.001
Platelet (%)	192.00 [124.50, 271.00]	135.50 [68.53, 218.00]	<0.001
Serum creatinine (μmol/L)	65.00 [48.00, 101.14]	98.50 [60.50, 203.80]	<0.001
Blood urea nitrogen (mmol/L)	7.76 [5.19, 12.00]	13.08 [8.30, 20.50]	<0.001
Serum albumin (g/L)	32.20 [28.99, 36.00]	31.20 [28.08, 34.52]	0.001
Total bilirubin	12.50 [7.84, 21.22]	17.79 [9.80, 37.10]	<0.001
ALT (U/L)	25.06(14.00,52.81)	26.85(14.00, 56.45)	0.42
GGT (U/L)	32.00(19.00,56.00)	44.27(23.60, 83.00)	0.002
C-reactive protein (mg/L)	43.40 [15.59, 101.35]	91.80 [38.40, 159.80]	<0.001
Procalcitonin (ng/mL)	0.32 [0.10, 1.58]	1.53 [0.46, 5.45]	<0.001
Lactate (mmol/L)	1.40 [0.90, 1.90]	1.60 [1.20, 2.60]	<0.001
Metabolic indices			
Blood Glucose (mmol/L)	8.15 [6.50, 10.50]	8.80 [6.50, 11.50]	0.135
Low-density lipoprotein (mmol/L)	1.87 [1.28, 2.51]	1.42 [0.88, 2.04]	<0.001
Triglyceride (mmol/L)	1.33 [0.93, 1.92]	1.55 [1.00, 2.40]	0.005
High density lipoprotein (mmol/L)	0.70 [0.51, 0.95]	0.54 [0.33, 0.88]	<0.001
Total cholesterol (mmol/L)	3.17 [2.41, 4.00]	2.63 [1.93, 3.43]	<0.001
TyG Index	9.07 [8.64, 9.53]	9.31 [8.75, 9.75]	0.001
TyHGB Index	10.39 [8.69, 13.07]	11.78 [9.62, 15.45]	<0.001

Abbreviation: APACHE II (Acute physiology and chronic health evaluation): SOFA (Sequential Organ Failure Assessment), NRS (nutrition risk screening), ALT (Alanine aminotransferase), GGT (Gamma-glutamyl transferase).

In terms of renal and hepatic function and inflammatory markers, survivors exhibited significantly lower levels of serum creatinine (65 vs 98.5 μmol/L), blood urea nitrogen (7.76 vs 13.08 mmol/L), γ-glutamyl transferase (32.00 vs 44.27 U/L), C-reactive protein (43.40 vs 91.8 mg/L), procalcitonin (0.32 vs 1.53 ng/mL) and lactate (1.40 vs 1.60 mmol/L) compared with non-survivors (all P < 0.01; Table 1).

In terms of metabolic biomarkers, both TyG index (9.07 vs 9.31, p = 0.001) and TyHGB (10.39 vs 11.78, p < 0.001) exhibited significant differences between survivors and non-survivors. In addition, survivors exhibited significantly higher levels of low-density lipoprotein (LDL) (1.87 vs 1.42 mmol/L), high-density lipoprotein (HDL) levels (0.70 vs 0.54 mmol/L) and total cholesterol (3.17 vs 2.63 mmol/L), along with lower triglyceride (TG) (1.33 vs 1.55 mmol/L) (all P < 0.01). No significant difference in glucose level was observed (8.15 vs 8.80, P = 0.135).

The distribution of TyHGB in the study population is summarized in Supplementary Table S3. The median TyHGB was 10.64 (IQR: 8.83–13.65), with values ranging from 5.69 to 42.47 in imputed samples.

### Restricted cubic spline analysis of TyHGB and 28‑day mortality

The association between the TyHGB index and 28-day mortality remained statistically significant across all risk-adjusted models (Table 2, Fig. 1, Fig. 2). To evaluate the potential non-linear dose-response relationship, restricted cubic spline (RCS) analysis was performed with 3 knots, using the median TyHGB value (10.64) as the reference. When comparing the 75th percentile to the 25th percentile of TyHGB, a higher index was significantly associated with increased 28-day mortality across all models. In Model 1 (adjusted for age, gender, and diabetes), the odds ratio (OR) comparing the 75th vs. 25th percentile was 1.65 (95% CI: 1.23−2.21, P < 0.001). After further adjusting for disease severity and organ dysfunction biomarkers in Model 2 (APACHE II score, lactate, total bilirubin, albumin and serum creatinine), the elevated risk remained statistically significant (OR = 1.40, 95% CI: 1.03−1.89, P = 0.009). Crucially, in the sensitivity analysis model (Model 3) which additionally incorporated clinical interventions (insulin, enteral/parenteral nutrition, corticosteroids), TyHGB maintained its independent prognostic value, with an OR of 1.44 (95% CI: 1.05−1.96, P = 0.011) for the 75th vs. 25th percentile comparison. Of note, the non-linear component of TyHGB was not statistically significant in all models (P for non-linearity > 0.05). Complete-case analysis yielded consistent results (Supplementary Table S4).

**Table 2:** Association between TyHGB and 28-day mortality in restricted cubic spline analysis.

	OR (95% CI)	P value	P for non-linearity
Model 1	1.65 (1.23−2.21)	< 0.001	0.168
Model 2	1.40 (1.03−1.89)	0.009	0.317
Model 3	1.44 (1.05−1.96)	0.011	0.233

Model 1 Age + Gender + Diabetes.

Model 2 Model 1 + APACHE II + Lactate + Total Bilirubin + Albumin + Serum Creatinine.

Model 3 Model 2 + Insulin use + PN + EN + Corticosteroids + Pre-index ICU length of stay.

**Fig. 1:**
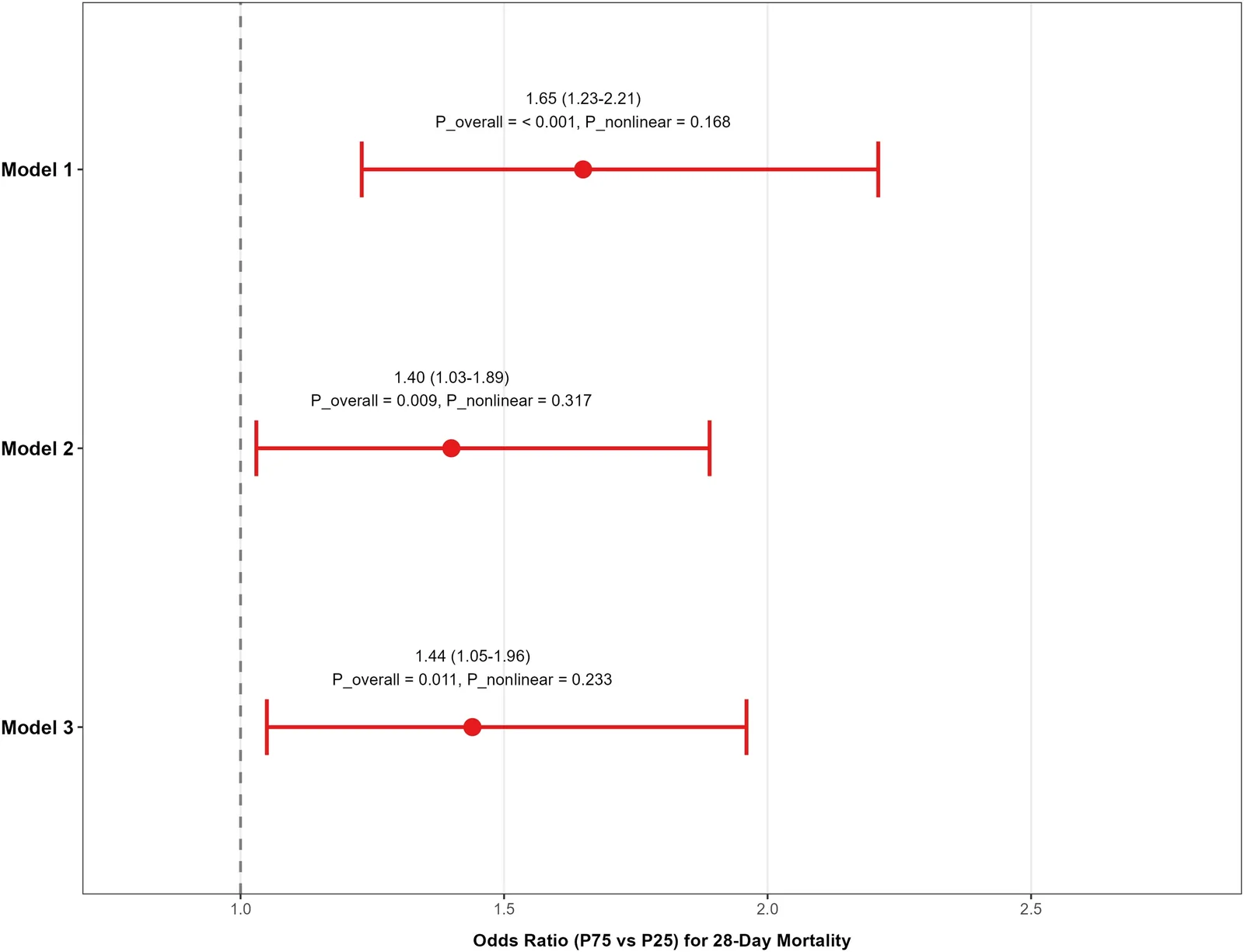
Forest plot for association between TyHGB and 28-day mortality.

**Fig. 2:**
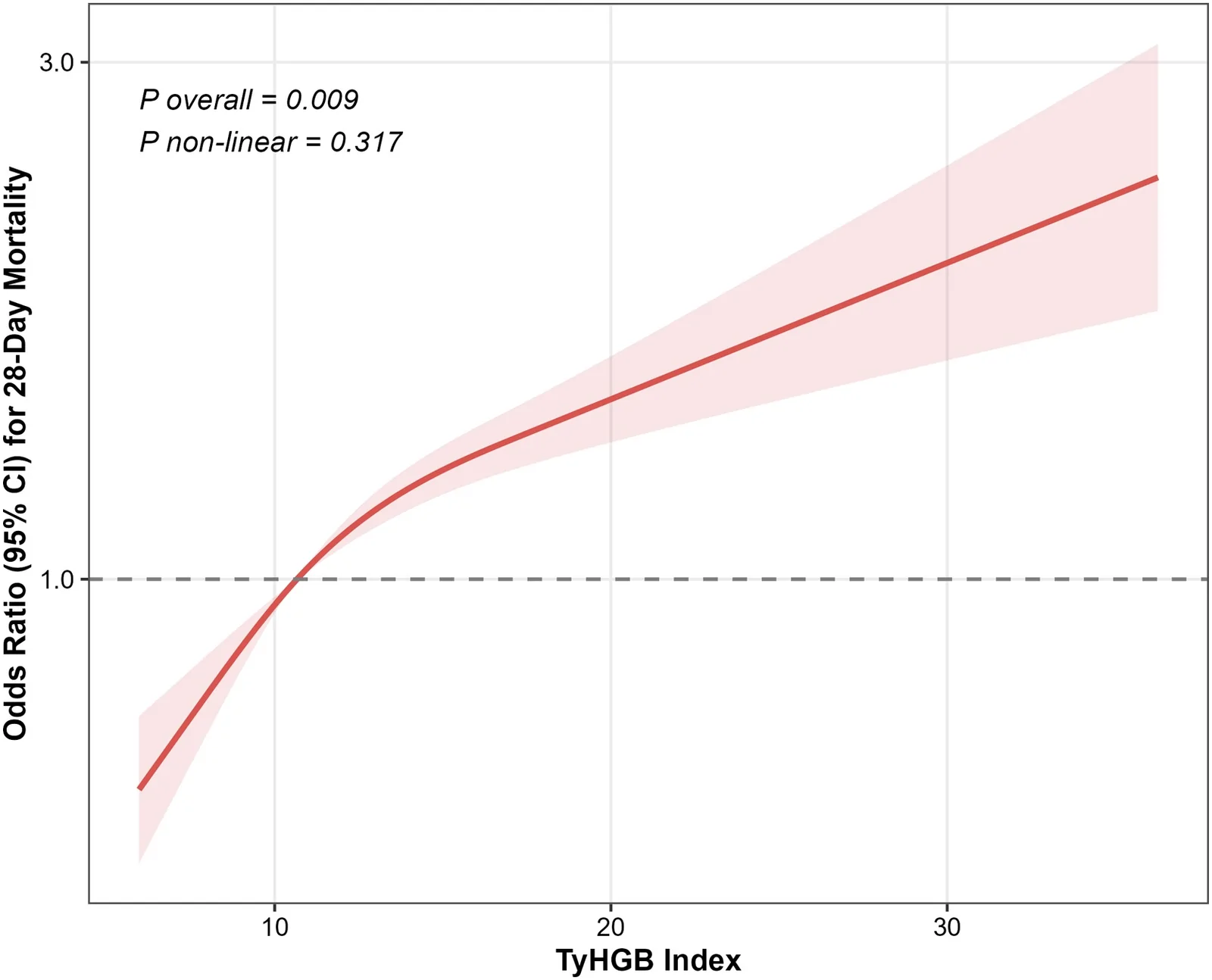
Dose-response relationship between TyHGB and 28-day mortality.

### Subgroup analysis

We further examined whether the relationship between TyHGB and 28-day mortality was consistent across subgroups defined by gender, age, APACHE II score, diabetes status, cerebrovascular disease and coronary heart disease using logistic regression analysis as linear association was detected according to previous RCS analysis (Table 3, Fig. 3).

**Table 3:** Subgroup analysis of the associations between TyHGB and 28-day mortality.

	Stratification	Events (N)	Mortality Rate (%)	OR (95% CI)	P Value	P for Interaction
Age	>=65 years old	764 (132)	17.3	1.05 (1.004−1.090)	0.033	0.910
	<65 years old	885 (93)	10.5	1.04 (0.998−1.072)	0.061	
Gender	Male	1077 (142)	13.2	1.05 (1.02−1.09)	0.002	0.335
	Female	572 (83)	14.5	1.02 (0.97−1.08)	0.393	
APACHE II Score	>=15	828 (176)	21.3	1.05 (1.01−1.08)	0.006	0.700
	<15	821 (49)	6.0	1.04 (0.99−1.10)	0.133	
Diabetes Mellitus	Yes	401 (67)	16.7	1.11 (1.04−1.19)	0.002	0.011
	No	1248 (158)	12.7	1.02 (0.99−1.06)	0.189	
CHD	Yes	562 (96)	17.1	1.05 (0.999−1.10)	0.053	0.873
	No	1087 (129)	11.9	1.04 (1.01−1.08)	0.012	
CVD	Yes	387 (56)	14.5	1.10 (1.03−1.18)	0.006	0.070
	No	1262 (169)	13.4	1.03 (1.001−1.06)	0.043	

Abbreviation: CHD (coronary heart disease), CVD (cerebrovascular disease).

Adjusted for variables in Model 2.

**Fig. 3:**
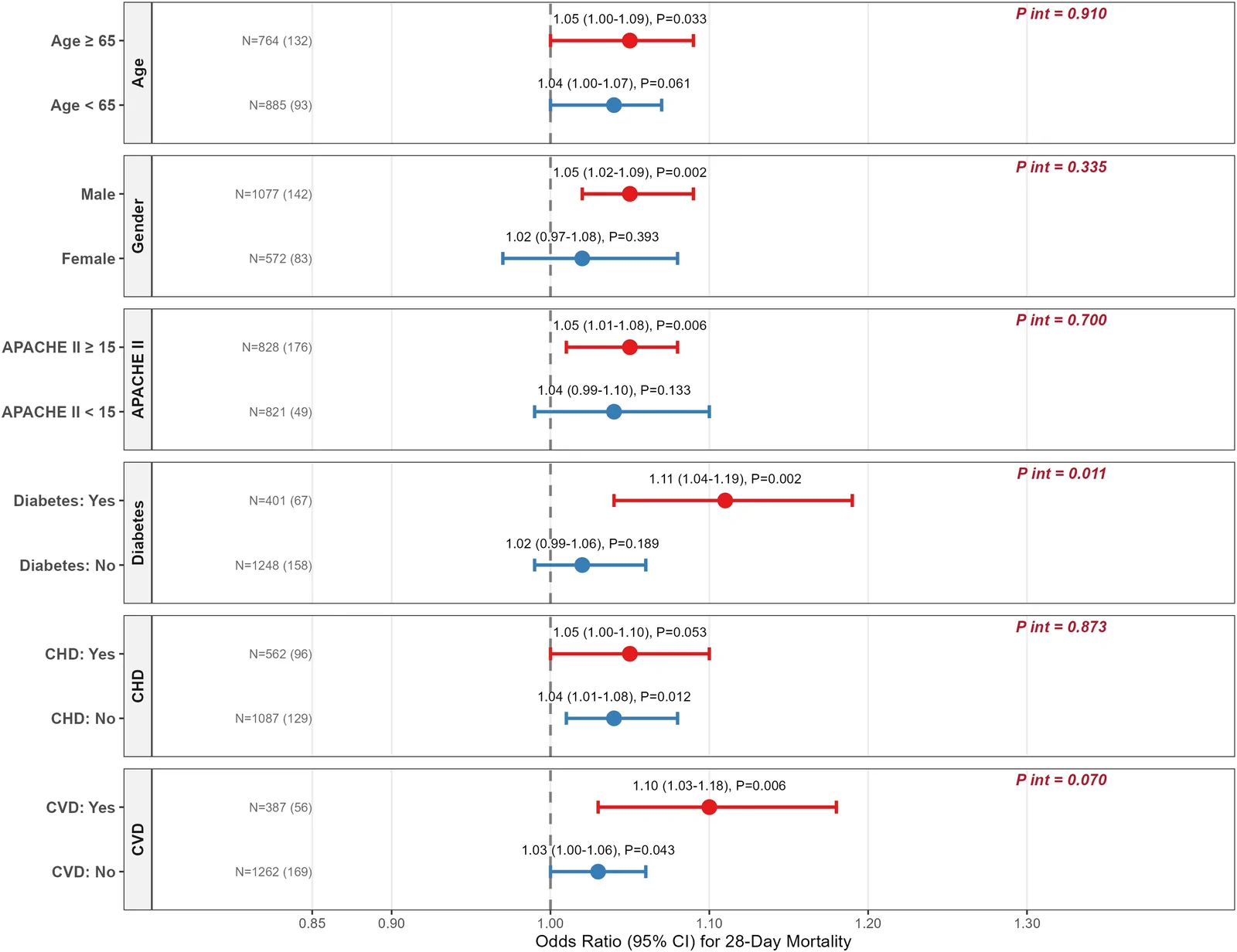
Forest plot for subgroup analyses of TyHGB with 28-day mortality.

The association between TyHGB and 28-day mortality was generally consistent across most subgroups. Notably, a nominally significant interaction was observed for diabetes mellitus (P for interaction = 0.011).

### Model performance and incremental predictive value

To evaluate the incremental predictive value of the metabolic indices for 28-day mortality, TyG, TyHGB and its components were individually added to the baseline prediction model (Table 4, Supplementary Table S6). In the overall population, TyHGB demonstrated a statistically significant improvement in AUC compared with the base model (ΔAUC = 0.014; AUC = 0.745; P = 0.043), with the highest AUC among all candidate models and the lowest AIC (1187.22) and BIC (1225.07) (Fig. 4). Notably, compared with the Joint model, TyHGB provided comparable or slightly better discrimination with fewer parameters, as reflected by the AIC and BIC values. Furthermore, incorporating TyHGB significantly improved overall model fit (LRT P < 0.001).

**Table 4:** Comparison of predictive performance of different models.

Population / Model	Apparent AUC (95% CI)	ΔAUC	P value	LRT	Brier Score	AIC	BIC
Overall Population							
Model A (Baseline)	0.731 (0.696−0.763)	—	—	—	0.109	1202.32	1234.77
HDL	0.738 (0.701−0.772)	0.007	0.398	<0.001	0.107	1188.26	1226.12
TG	0.734 (0.699−0.766)	0.003	0.346	0.076	0.109	1201.18	1239.03
GLU	0.731 (0.696−0.763)	0.000	0.953	0.627	0.109	1204.09	1241.94
BMI	0.733 (0.698−0.765)	0.002	0.521	0.181	0.109	1202.53	1240.39
Joint	0.740 (0.703−0.774)	0.009	0.321	<0.001	0.106	1190.58	1244.66
Model B (+TyG)	0.736 (0.701−0.768)	0.005	0.303	0.010	0.108	1197.66	1235.52
Model C (+TyHGB)	0.745 (0.710−0.777)	0.014	0.043	<0.001	0.107	1187.22	1225.07
Diabetic Subgroup							
Model A (Baseline)	0.673 (0.595−0.743)	—	—	—	0.130	347.87	367.84
HDL	0.711 (0.633−0.779)	0.038	0.150	0.001	0.125	339.13	363.09
TG	0.681 (0.602−0.750)	0.008	0.446	0.256	0.130	348.58	372.55
GLU	0.679 (0.602−0.746)	0.006	0.583	0.244	0.130	348.52	372.48
BMI	0.678 (0.597−0.750)	0.005	0.731	0.137	0.128	347.66	371.63
Joint	0.720 (0.639−0.788)	0.046	0.117	0.003	0.123	340.02	375.96
Model B (+TyG)	0.692 (0.616−0.759)	0.019	0.259	0.031	0.128	345.22	369.18
Model C (+TyHGB)	0.723 (0.648−0.787)	0.050	0.049	<0.001	0.123	332.9	356.86
Non-Diabetic Subgroup							
Model A (Baseline)	0.747 (0.707−0.784)	—	—	—	0.102	859.07	884.71
HDL	0.749 (0.706−0.786)	0.001	0.861	0.007	0.100	853.71	884.49
TG	0.750 (0.710−0.786)	0.003	0.367	0.184	0.101	859.31	890.08
GLU	0.747 (0.707−0.784)	0.000	0.943	0.527	0.101	860.67	891.44
BMI	0.749 (0.709−0.785)	0.002	0.357	0.531	0.102	860.67	891.45
Joint	0.751 (0.709−0.789)	0.004	0.665	0.047	0.100	857.42	903.58
Model B (+TyG)	0.751 (0.711−0.787)	0.003	0.408	0.112	0.101	858.54	889.31
Model C (+TyHGB)	0.756 (0.716−0.792)	0.009	0.152	0.015	0.101	855.2	885.98

P value is generated by Delong test; Abbreviation: AUC, area under the receiver operating characteristic curve; CI, confidence interval; HDL, high-density lipoprotein cholesterol; TG, triglycerides; BMI, body mass index.

Model A (Baseline): age + gender + APACHE II + lactate + diabetes.

Model B: Baseline + TyG.

Model C: Baseline + TyHGB.

Joint: Baseline + all four components (HDL + TG + BMI + glucose).

**Fig. 4:**
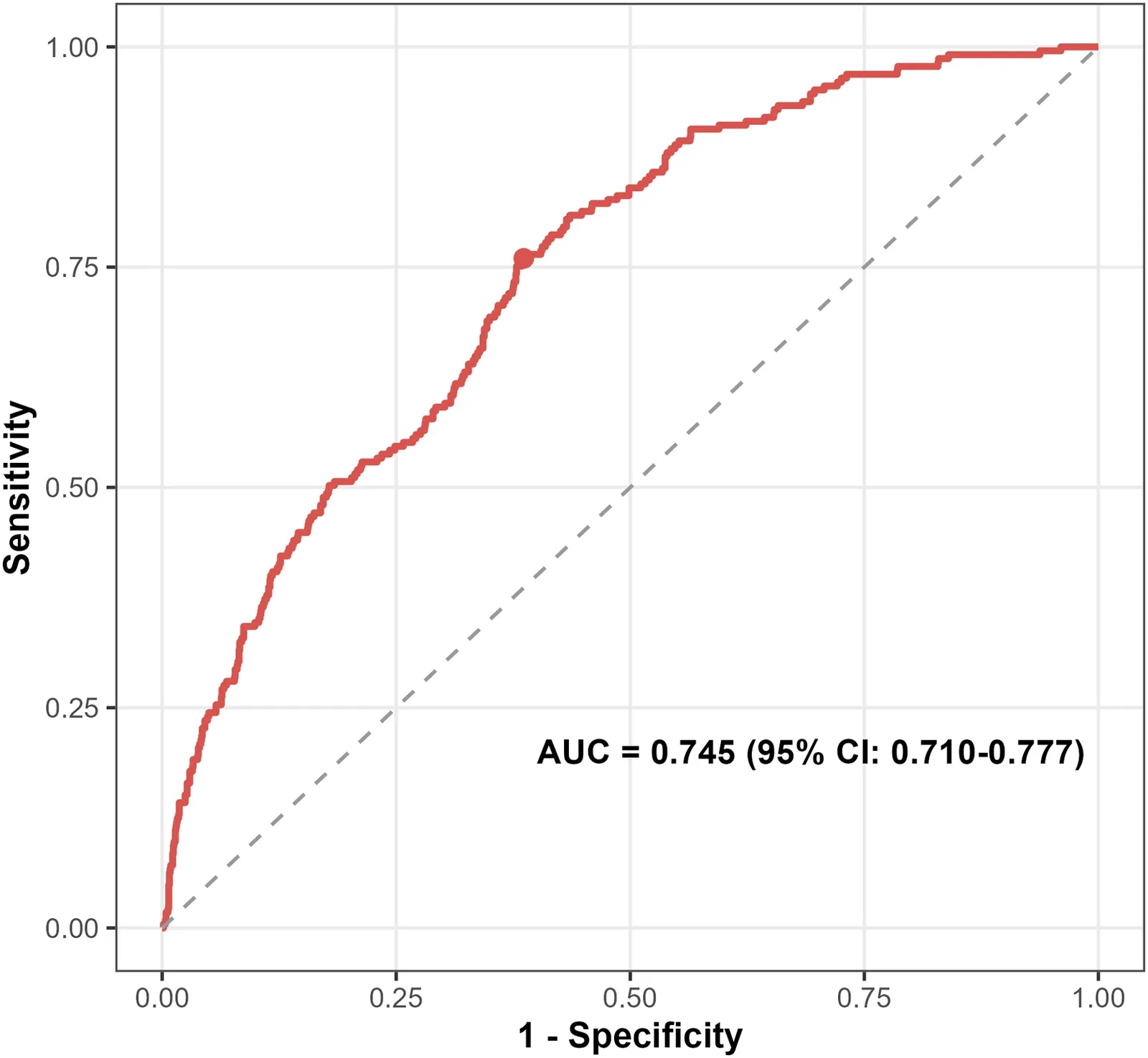
The ROC curve of TyHGB as an index for predicting 28-day mortality.

Guided by the significant interaction effect observed for diabetes mellitus (P for interaction = 0.011), we further conducted exploratory subgroup analyses stratified by diabetes status (Table 4). In the diabetic subgroup, the base model exhibited limited discrimination (AUC = 0.673, 95% CI: 0.595−0.743). Incorporating TyHGB substantially improved discrimination, with ΔAUC of 0.050 (AUC = 0.723, 95% CI: 0.648−0.787; P = 0.049). Still, TyHGB achieved the highest predictive performance among all candidate models in diabetic patients, supported by a highly significant LRT (P < 0.001) and the lowest AIC (332.9) and BIC (356.86).

Internal validation using bootstrap optimism-correction demonstrated that adding TyHGB to the baseline model improved the optimism-corrected AUC from 0.725 to 0.737, with well-calibrated slopes close to 1.0 (0.961 and 0.956, respectively) and an increase in R² from 0.121 to 0.137 (Table 5). In the overall cohort, TyHGB significantly improved risk reclassification as measured by the continuous NRI (0.328, 95% CI: 0.177−0.479, P < 0.001) and IDI (0.012, 95% CI: 0.003−0.022, P = 0.009). The improvement remained significant in diabetic patients (NRI = 0.515, 95% CI: 0.230−0.801, P < 0.001; IDI = 0.041, 95% CI: 0.006−0.076, P = 0.022), whereas in non-diabetic patients the IDI did not reach statistical significance (IDI = 0.006, 95% CI: −0.002−0.013, P = 0.150). Besides, the DCA curve for the diabetic subgroup showed that Model C consistently outperformed Models A and B across the 10 %–30 % threshold range (Supplementary Table S5). Still, this finding should be considered exploratory and requires further validation.

**Table 5:** Performance and internal validation of the TyHGB mortality prediction model.

Metric	Overall: Model A	Overall: Model C	Diabetic: Model A	Diabetic: Model C	Non-Diabetic: Model A	Non-Diabetic: Model C
Discrimination & Fit						
Optimism-corrected AUC	0.725	0.737	0.653	0.703	0.741	0.747
Optimism-corrected R^2	0.121	0.137	0.072	0.122	0.131	0.138
Calibration & Accuracy						
Calibration Slope	0.961	0.956	0.901	0.894	0.960	0.951
Calibration Intercept	−0.064	−0.070	−0.141	−0.148	−0.063	−0.083
Optimism-corrected Brier Score	0.110	0.109	0.134	0.130	0.103	0.103
Reclassification Performance						
Continuous NRI (95% CI)	—	0.328 (0.177 to 0.479)	—	0.515 (0.230 to 0.801)	—	0.247 (0.069 to 0.425)
NRI P-value	—	<0.001	—	<0.001	—	0.007
IDI (95% CI)	—	0.012 (0.003 to 0.022)	—	0.041 (0.006 to 0.076)	—	0.006 (-0.002 to 0.013)
IDI P-value	—	0.009	—	0.022	—	0.150
Diagnostic & Classification Utilities						
Derived Cutoff	0.124	0.113	0.188	0.166	0.112	0.112
Sensitivity (95% CI)	73.4% (67.3%–79.4%)	78.8% (72.7%–84.9%)	55.6% (31.0%–80.1%)	67.8% (54.3%–81.3%)	75.8% (68.2%–83.5%)	76.0% (67.2%–84.8%)
Specificity (95% CI)	63.0% (60.2%–65.9%)	58.6% (54.7%–62.5%)	73.1% (51.0%–95.2%)	69.8% (61.7%–77.9%)	63.7% (58.7%–68.7%)	63.6% (57.0%–70.1%)
PPV (95% CI)	23.9% (20.7%–27.1%)	23.1% (20.0%–26.2%)	30.7% (18.5%–42.9%)	31.2% (23.0%–39.3%)	23.3% (19.4%–27.1%)	23.3% (19.3%–27.3%)
NPV (95% CI)	93.7% (92.2%–95.3%)	94.6% (93.0%–96.2%)	89.3% (85.0%–93.6%)	91.6% (87.9%–95.2%)	94.8% (93.1%–96.5%)	94.8% (93.1%–96.6%)

Internal validation performed using 500 bootstrap resamples for optimism correction and calibration.

Abbreviations: AUC, area under the curve; CI, confidence interval; NRI, net reclassification improvement; IDI, integrated discrimination improvement; PPV, positive predictive value; NPV, negative predictive value.

## Discussion

The present observational cohort study identified a significant and independent association between TyHGB and 28-day mortality in patients present in ICU at the landmark time in China. To our knowledge, this is the first study to evaluate the prognostic value of TyHGB in the intensive care setting. The association persisted after extensive covariate adjustment and TyHGB demonstrated a modest but statistically significant improvement in model discrimination in the overall cohort, with the lowest AIC and BIC among all candidate models, suggesting that TyHGB may provide additional prognostic information beyond traditional risk factors. Notably, a significant interaction was observed with diabetes status (P = 0.011), and the improvement in discrimination was more pronounced in diabetic patients, suggesting that the association between TyHGB and mortality may be particularly relevant in metabolically vulnerable populations. Given the exploratory nature of these analyses, these findings require independent validation.

Unlike traditional biomarkers with established reference intervals, TyHGB does not currently have a universally accepted clinical reference range. Previous population-based studies have generally reported lower TyHGB distributions, with median values typically ranging around 7–10 [17,[21], [22], [23], [24]]. The higher TyHGB levels observed in our ICU cohort may reflect the combined metabolic alterations associated with acute critical illness, including stress hyperglycemia, insulin resistance and lipid remodeling. Therefore, mean/median TyHGB values around 10–12 in our cohort should be interpreted as reflecting the distribution of metabolic stress within this critically ill population rather than as a predefined clinical threshold.

The observed independent association between TyHGB and 28-day mortality is biologically plausible. Critical illness brings about systemic metabolic changes. Anabolic processes in the liver are accelerated through gluconeogenesis driven by lactate, amino acids and glycerol, while catabolism is concurrently activated in adipose tissue and skeletal muscle to provide necessary substrates for the anabolism [7]. The abnormal co-occurrence of anabolism and catabolism, coupled with insulin resistance possibly driven by complex inflammatory responses [25,26], leads to excessive glucose accumulation and futile substrate cycling. Concurrently, lipid metabolism is similarly dysregulated via two mechanisms. First, lipolysis is accelerated through both a canonical pathway (stress hormones coupled with insulin resistance) and a non-canonical pathway (inflammatory cytokines directly activating adipocyte lipase) [27,28]. Second, fatty acid utilization is impaired, as evidenced by elevated plasma acylcarnitines in non-surviving septic patients and reduced PPAR-α activity in liver biopsies from non-surviving critically ill patients [29,30].

The clinical consequences of this global metabolic dysregulation are substantial. Stress-induced hyperglycemia and insulin resistance have been independently associated with increased risks of nosocomial infections, endothelial dysfunction and mortality, although randomized trials have failed to demonstrate survival benefit from intensive glucose control alone as aforementioned [31,32]. Concurrent muscle wasting contributes to ICU-acquired weakness, prolonged mechanical ventilation, and extended hospital stays [33,34]. Inverse association between systemic cholesterol and mortality was identified in sepsis and other critical illnesses (with HDL showing the highest reliability), a phenomenon described as a maladaptive regulatory shift rather than simple dysregulation [5]. The composite advantage is strengthened by genomic evidence showing that 62 independent loci harbor significantly stronger associations with the TG/HDL ratio than with TG or HDL in isolation, directly mapping to core insulin resistance mechanisms [35,36]. Collectively, TyHGB integrates glycemic stress, adiposity and dyslipidemia to provide a multidimensional assessment of host vulnerability.

In our cohort, HDL and TG showed more pronounced disparities between survivors and non-survivors than static blood glucose or BMI. This indicates that acute inflammation-induced lipid dysregulation and impaired lipoprotein clearance are indeed major contributors to the observed risk in critically ill patients. However, the key advantage of TyHGB over single components lies in its capacity to capture systemic metabolic vulnerability rather than isolated biochemical abnormalities. In critical care, static glucose levels are frequently flattened by active clinical management and single BMI measurements are often distorted by post-resuscitation fluid overload. When analyzed in isolation, these parameters may lose their prognostic sensitivity. Besides, it is well recognized that both glucose level and BMI are associated with TG and HDL [37,38]. Within the TyHGB framework, glucose and BMI function as contextual calibrators for glycemic stress and physical baseline, respectively. This holistic approach provides a potential explanation for why composite metabolic indices may capture aspects of metabolic vulnerability that are not fully represented by individual components.

TyHGB modestly but significantly improved discrimination in the overall cohort (ΔAUC = +0.014, P = 0.043), with consistent improvements in model fit (LRT P < 0.001), optimism-corrected AUC (0.725 to 0.737) and risk reclassification (NRI = 0.328, P < 0.001; IDI = 0.012, P = 0.009). TyG did not significantly improve discrimination (ΔAUC = +0.005, P = 0.303). In diabetic patients, the improvement was more pronounced (ΔAUC = +0.050, P = 0.049; IDI = 0.041, P = 0.022; NRI = 0.515, P < 0.001), consistent with a significant interaction (P = 0.011). These exploratory findings require independent validation. Prior studies have established the close link between TyHGB and diabetes-related complications [17,20,24]. Diabetic patients are often susceptible to worse outcomes in responses to acute critical stress due to pre-existing metabolic remodeling [39,40]. This may be attributable to the fact that diabetes represents a state in which multiple components incorporated into TyHGB, including insulin resistance, hyperglycemia and dyslipidemia, are chronically altered. Thus, standard severity scores primarily quantify acute physiological derangements but may not fully capture chronic metabolic vulnerability. By integrating these intertwined metabolic domains into a unified score, TyHGB may offer potential exploratory value for risk stratification in diabetic patients. Nevertheless, given the data-driven nature of this subgroup analysis, these findings should be regarded as hypothesis-generating and require formal prospective validation in dedicated cohorts.

Several strengths of this study should be acknowledged. First, to our knowledge, this is one of the largest multicenter cohort studies across 113 ICUs in mainland China evaluating the metabolic marker TyHGB under a landmark design. Second, TyHGB demonstrated modest improvements in model fit and risk reclassification metrics in the overall cohort. Notably, a potential signal was observed in the diabetic subgroup, but given the exploratory nature of this analysis and the absence of multiplicity adjustment, this finding should be interpreted cautiously and requires independent validation. Third, since TyHGB is derived from routinely available clinical parameters, it offers a low-cost, easily accessible candidate metric for metabolic risk stratification, providing hypothesis-generating insights for future research.

Several limitations should be acknowledged. First, due to the observational design, residual confounding from unmeasured variables cannot be entirely excluded. Second, despite the adjustment for pre-index ICU stay, the use of point-prevalence sampling at the index date brings length-related selection. The study population therefore represents the prevalent ICU population at the index time, thus our findings should not be generalised to the broader population of critically ill patients. Third, TyHGB was calculated from a single laboratory measurement and therefore could not capture dynamic changes. In addition, metabolic interventions and intensive care therapies during the ICU stay, such as continuous renal replacement therapy and propofol sedation, may have dynamically altered blood glucose and lipid profiles, which could have influenced TyHGB levels. Fourth, potential heterogeneity in laboratory measurement methodologies across different participating centers could not be completely eliminated. Fifth, deriving and evaluating the optimal cutoff value within the same single cohort may have introduced potential optimism or overfitting. Internal validation using bootstrap was performed to mitigate this. Sixth, the diabetes subgroup finding was exploratory, and given that multiple subgroup comparisons were performed without adjustment for multiplicity, this result should be interpreted cautiously and requires independent confirmation. Finally, because our cohort was recruited exclusively from Chinese ICUs, the generalizability of our findings to other geographical populations and healthcare settings remains to be confirmed.

## Conclusions

Among patients present in participating ICUs at the landmark time, higher TyHGB was associated with subsequent 28-day mortality. However, adding TyHGB to the baseline risk prediction model resulted in only small improvements in overall predictive performance. The exploratory findings in patients with diabetes require independent validation.

## Author contributions

Formal anlaysis: XJP, ZLW, HL. Writing: XJP, YMX, HXD. Visualization: YMX ZLW. Investigation: HXD, CCY, SZ, SSH, YZC, LDZ. Data curation: HL, ZWL, SSH, YZC, LDZ. Project administration: TW, YAL. Supervision: JL, DCC, SZ. All authors critically revised the manuscript and approved the final version.

## Fundings

This work was supported by the National Natural Science Foundation of China ( No. 82302477, No. 82572500), the Fundamental Research Funds for the Central Universities (YG2023LC01) and Shanghai Municipal Health Commission Clinical Research Special Project (No. 202340126).

## Additional information

All the authors declare that they do not have conflicts of interest.

## Data availability statement

The data that support the findings of this survey are available from the corresponding author upon reasonable request.

## Declaration of competing interest

The authors declare that they have no known competing financial interests or personal relationships that could have appeared to influence the work reported in this paper.
