# Supplementary material for: Association between triglyceride high-density cholesterol-glucose body index and 28-day mortality in critically ill patients: a multicenter observational study in China
Source: Ann Intensive Care. 2026 Sep 30;16:100173. doi: 10.1016/j.aicoj.2026.100173 (PMC13717743; doi:10.1016/j.aicoj.2026.100173)
Supplement: Supplementary file 1 [file mmc1.docx]

| Variable | Missing (n) | Missing (%) |
| --- | --- | --- |
| HDL | 297 | 18.01 |
| TG | 286 | 17.34 |
| Glucose | 166 | 10.07 |
| Lactate | 75 | 4.55 |
| APACHE II | 50 | 3.03 |
| Albumin | 49 | 2.97 |
| Total bilirubin | 39 | 2.37 |
| Creatinine | 32 | 1.94 |
| Death | 0 | 0.00 |
| Age | 0 | 0.00 |
| Gender | 0 | 0.00 |
| Diabetes | 0 | 0.00 |
| Insulin | 0 | 0.00 |
| Parenteral Nutrition | 0 | 0.00 |
| Enteral Nutrition | 0 | 0.00 |
| Corticosteroids | 0 | 0.00 |
| BMI | 0 | 0.00 |
| Pre-index ICU length of stay | 0 | 0.00 |

Supplementary Table S1 Missing rate of variables

Supplementary Table S2 Baseline characteristics of complete-case cohort vs. imputed cohort

| Variable | Complete-case (N = 1,190) | Pooled Imputed (N = 1,649) | SMD |
| --- | --- | --- | --- |
| **Demographics** |  |  |  |
| Age, mean ± SD / median (IQR) | 59.83 ± 15.71 / 62.00 (50.00-72.00) | 60.58 ± 15.74 / 63.00 (51.00-72.00) | 0.048 |
| Height, mean ± SD / median (IQR) | 166.48 ± 8.94 / 168.00 (160.00-173.00) | 166.39 ± 9.47 / 168.00 (160.00-173.00) | 0.010 |
| Weight, mean ± SD / median (IQR) | 64.82 ± 13.62 / 65.00 (55.00-70.88) | 64.38 ± 13.68 / 65.00 (55.00-70.00) | 0.032 |
| BMI, mean ± SD / median (IQR) | 23.20 ± 3.84 / 22.95 (20.76-25.10) | 23.03 ± 3.78 / 22.86 (20.66-24.97) | 0.044 |
| Male, n (%) | 769 (64.6) | 1,077 (65.3) | 0.014 |
| **Source, n (%)** |  |  |  |
| Emergency | 493 (41.5) | 646 (39.2) | 0.046 |
| Internal Medicine | 279 (23.5) | 428 (26.0) | 0.058 |
| Surgery | 417 (35.1) | 573 (34.8) | 0.006 |
| **Comorbidities, n (%)** |  |  |  |
| Diabetes | 285 (23.9) | 401 (24.3) | 0.009 |
| Cardiovascular disease | 401 (33.7) | 562 (34.1) | 0.008 |
|  |  |  |  |
| Cerebrovascular disease | 260 (21.8) | 387 (23.5) | 0.039 |
| Respiratory disease | 123 (10.3) | 188 (11.4) | 0.034 |
| Tumor | 114 (9.6) | 180 (10.9) | 0.044 |
| Hematologic disease | 36 (3.0) | 47 (2.9) | 0.010 |
| **Severity scores** |  |  |  |
| APACHE II, mean ± SD / median (IQR) | 15.00 ± 7.53 / 14.00 (10.00-20.00) | 15.11 ± 7.79 / 14.84 (10.00-20.00) | 0.015 |
| SOFA, mean ± SD / median (IQR) | 6.08 ± 4.24 / 5.50 (3.00-8.00) | 6.02 ± 4.42 / 5.00 (3.00-8.00) | 0.014 |
| NRS-2002, mean ± SD / median (IQR) | 3.68 ± 1.99 / 3.00 (3.00-5.00) | 3.69 ± 2.07 / 3.00 (3.00-5.00) | 0.007 |
| Charlson Comorbidity Index, mean ± SD / median (IQR) | 2.37 ± 2.29 / 2.00 (1.00-3.00) | 2.43 ± 2.29 / 2.00 (1.00-3.00) | 0.027 |
| **Laboratory parameters** |  |  |  |
| WBC (×10⁹/L), mean ± SD / median (IQR) | 12.23 ± 26.27 / 9.80 (6.99-13.52) | 13.80 ± 60.51 / 9.80 (7.19-13.60) | 0.034 |
| Hemoglobin (g/L), mean ± SD / median (IQR) | 95.65 ± 31.93 / 92.00 (78.00-109.00) | 96.86 ± 34.14 / 94.00 (79.00-110.00) | 0.036 |
| Platelet (×10⁹/L), mean ± SD / median (IQR) | 204.59 ± 127.51 / 182.00 (114.25-269.75) | 203.01 ± 124.27 / 185.00 (116.00-264.50) | 0.013 |
| Creatinine (µmol/L), mean ± SD / median (IQR) | 114.31 ± 134.12 / 68.25 (48.00-115.00) | 114.25 ± 137.52 / 67.74 (48.78-115.74) | 0.000 |
| BUN (mmol/L), mean ± SD / median (IQR) | 15.77 ± 89.75 / 8.43 (5.45-13.66) | 14.71 ± 78.40 / 8.10 (5.30-13.44) | 0.013 |
| Albumin (g/L), mean ± SD / median (IQR) | 32.37 ± 5.87 / 32.00 (28.80-35.48) | 32.48 ± 5.91 / 32.00 (28.80-35.97) | 0.019 |
| Total bilirubin (µmol/L), mean ± SD / median (IQR) | 25.80 ± 45.51 / 13.47 (8.52-23.20) | 24.91 ± 45.05 / 12.98 (7.99-22.58) | 0.020 |
| ALT (U/L), mean ± SD / median (IQR) | 63.40 ± 190.42 / 26.00 (14.88-54.00) | 63.78 ± 204.91 / 25.50 (14.00-53.00) | 0.002 |
| GGT (U/L), mean ± SD / median (IQR) | 85.48 ± 122.07 / 48.80 (25.00-97.25) | 82.26 ± 117.56 / 45.00 (24.80-94.66) | 0.027 |
| CRP (mg/L), mean ± SD / median (IQR) | 78.44 ± 98.81 / 54.50 (17.35-113.00) | 75.55 ± 92.05 / 51.00 (17.00-111.43) | 0.030 |
| PCT (ng/mL), mean ± SD / median (IQR) | 5.84 ± 27.11 / 0.43 (0.12-2.08) | 5.52 ± 25.02 / 0.41 (0.12-1.99) | 0.012 |
| Lactate (mmol/L), mean ± SD / median (IQR) | 1.74 ± 1.56 / 1.40 (0.90-2.00) | 1.81 ± 1.87 / 1.40 (0.99-2.00) | 0.041 |
| Glucose (mmol/L), mean ± SD / median (IQR) | 8.81 ± 3.74 / 8.00 (6.50-10.30) | 8.96 ± 3.71 / 8.17 (6.50-10.64) | 0.039 |
| LDL (mmol/L), mean ± SD / median (IQR) | 2.21 ± 7.19 / 1.81 (1.23-2.48) | 2.32 ± 7.84 / 1.81 (1.23-2.46) | 0.015 |
| **Lipid and metabolic parameters** |  |  |  |
| Triglycerides (mmol/L), mean ± SD / median (IQR) | 1.81 ± 3.58 / 1.36 (0.94-2.00) | 1.85 ± 3.66 / 1.35 (0.94-1.99) | 0.009 |
| HDL (mmol/L), mean ± SD / median (IQR) | 0.79 ± 2.11 / 0.67 (0.46-0.93) | 0.88 ± 3.19 / 0.68 (0.47-0.94) | 0.036 |
| Total cholesterol (mmol/L), mean ± SD / median (IQR) | 3.41 ± 5.96 / 3.08 (2.35-3.98) | 3.39 ± 5.59 / 3.09 (2.33-3.98) | 0.004 |
| TyG, mean ± SD / median (IQR) | 9.14 ± 0.72 / 9.09 (8.66-9.57) | 9.15 ± 0.72 / 9.11 (8.67-9.58) | 0.017 |
| TyHGB, mean ± SD / median (IQR) | 12.00 ± 5.33 / 10.53 (8.77-13.64) | 12.03 ± 5.31 / 10.64 (8.83-13.65) | 0.006 |
| **Outcome** |  |  |  |
| Death, n (%) | 166 (13.9) | 225 (13.6) | 0.009 |

SD, standard deviation; IQR, interquartile range; SMD, standardized mean difference; BMI, body mass index; APACHE II, Acute Physiology and Chronic Health Evaluation II; SOFA, Sequential Organ Failure Assessment; NRS-2002, Nutritional Risk Screening 2002; WBC, white blood cell; BUN, blood urea nitrogen; ALT, alanine aminotransferase; GGT, gamma-glutamyl transferase; CRP, C-reactive protein; PCT, procalcitonin; LDL, low-density lipoprotein; HDL, high-density lipoprotein; TyG, triglyceride-glucose index; TyHGB, triglyceride-glucose index combined with BMI and HDL.

Supplementary Table S3 Distribution of TyHGB in critically ill patients

| Metric | Complete-case (N = 1190) | Pooled Imputed (N = 1649) |
| --- | --- | --- |
| Sample Size (N) | 1190 | 1649 |
| Mean ± SD | 12.00 ± 5.33 | 12.03 ± 5.31 |
| Median (IQR) | 10.53 (8.77-13.64) | 10.64 (8.83-13.65) |
| Range (Min-Max) | 5.70-42.00 | 5.69-42.47 |
| Quartile 1 (Q1: ≤ 25th) | ≤ 8.77 | ≤ 8.83 |
| Quartile 2 (Q2: 25th-50th) | 8.77-10.53 | 8.83-10.64 |
| Quartile 3 (Q3: 50th-75th) | 10.53-13.64 | 10.64-13.65 |
| Quartile 4 (Q4: > 75th) | > 13.64 | > 13.65 |

SD, standard deviation; IQR, interquartile range

Supplementary Table S4 Complete-Case Analysis (N=1190)

| Model | TyHGB OR (95% CI) | P value | Nonlinear P |
| --- | --- | --- | --- |
| Model 1 | 1.79 (1.28-2.50) | < 0.001 | 0.120 |
| Model 2 | 1.52 (1.08-2.14) | 0.002 | 0.279 |
| Model 3 | 1.52 (1.07-2.16) | 0.004 | 0.272 |

Model 1 age + gender + diabetes

Model 2 Model 1+ APACHE II + Lactate + Total Bilirubin + Albumin + Serum Creatinine

Model 3 Model 2 + Insulin use + PN + EN + corticosteroids + pre-index ICU length of stay

Supplementary Table S5 Net Benefit at Selected Thresholds (Diabetes Subgroup)

| Threshold | Model A | Model B | Model C |
| --- | --- | --- | --- |
| 10% | 0.0639 | 0.0640 | 0.0660 |
| 20% | 0.0232 | 0.0272 | 0.0277 |
| 30% | 0.0093 | 0.0050 | 0.0123 |

### Supplementary Table S6 Full Model Specification

|  | Overall population | | Diabetes | | Non-diabetes | |
| --- | --- | --- | --- | --- | --- | --- |
|  | OR (95% CI) | P | OR (95% CI) | P | OR (95% CI) | P |
| Age | 1.02 (1.01-1.03) | 0.001 | 1.02 (1.00-1.05) | 0.108 | 1.02 (1.01-1.03) | 0.002 |
| Gender | 0.92 (0.67-1.25) | 0.590 | 1.21 (0.67-2.17) | 0.529 | 0.84 (0.58-1.21) | 0.353 |
| APACHE II | 1.08 (1.06-1.10) | < 0.001 | 1.08 (1.04-1.12) | < 0.001 | 1.08 (1.06-1.11) | < 0.001 |
| Lactate | 1.10 (1.03-1.18) | 0.007 | 1.12 (0.93-1.37) | 0.238 | 1.10 (1.02-1.18) | 0.013 |
| Diabetes | 1.09 (0.78-1.52) | 0.625 | — | — | — | — |
| TyHGB | 1.05 (1.03-1.08) | < 0.001 | 1.12 (1.05-1.19) | 0.001 | 1.04 (1.01-1.07) | 0.020 |
